# Technical field measurements of muscular workload during stocking activities in supermarkets: cross-sectional study

**DOI:** 10.1038/s41598-022-04879-8

**Published:** 2022-01-18

**Authors:** Sebastian Venge Skovlund, Rúni Bláfoss, Sebastian Skals, Markus Due Jakobsen, Lars Louis Andersen

**Affiliations:** 1 National Research Centre for the Working Environment, Lersø Parkallé 105, 2100 Copenhagen, Denmark; 2 Research Unit for Muscle Physiology and Biomechanics, Department of Sports Science and Clinical Biomechanics, University of Southern Denmark, Odense, Denmark; 3 Sport Sciences-Performance and Technology, Department of Health Science and Technology, Aalborg University, Aalborg, Denmark

**Keywords:** Occupational health, Risk factors

## Abstract

Multiple studies have reported high prevalence of musculoskeletal disorders among supermarket workers. Technical field measurements can provide important knowledge about ergonomic risk factors for musculoskeletal disorders in the physical working environment, but these measurements are lacking in the supermarket sector. Therefore, using wearable electromyography and synchronous video recording in 75 supermarket workers, this cross-sectional study measured muscular workload during stocking activities in six different types of general store departments and during the thirteen most common work tasks across five different supermarket chains. Our results showed that muscular workload varies, especially for the low-back muscles, across (1) supermarket chains, (2) departments, and (3) specific stocking activities. Highest workloads of the low-back and neck/shoulders were seen in the fruit and vegetables department and during heavy, two-handed lifts of parcels (especially without using technical aids). In conclusion, physical work demands during supermarket stocking activities differ between chains, departments, and work tasks. These results can be used by company representatives and work environment professionals to specifically address and organize the stocking procedures to reduce the muscular workload during supermarket stocking.

## Introduction

High physical work demands including frequent and heavy lifting, twisting and bending of the trunk, and squatting and kneeling have been identified as significant risk factors for musculoskeletal disorders (MSD)^1–4^, long-term sickness absence^5–7^, reduced work ability^8^, disability pension^9–11^, and loss of paid employment^12^. However, 26–39% of long-term sickness absence among the general working population may be prevented by reducing the physical work demands, with larger preventive potential (~ 40–45%) among blue-collar workers^5,6^. Hence, identifying specific ergonomic risk factors in the physical working environment serves as an important first step in developing efficient preventive interventions.

Workers in supermarkets (or grocery stores) represent one job group with both high physical work demands and high prevalence of MSD, especially with regards to low-back (LBP) and neck/shoulder pain (NSP)^13–20^. Hence, preventive action is needed to reduce the physical demands of supermarket work.

In a societal perspective, the supermarket sector plays the central role of ensuring a constant supply of food, beverages and other staple goods. The supermarket sector is one of the largest work sectors in Denmark with approximately 70,000 employees across 3000 different stores (2017 numbers), including part time and full time workers in all age groups^21^. Supermarket stores vary greatly in size, but most stores are organized into functional departments with specialized work tasks^13,17,22^, e.g. cashiers and stockers. Still, all supermarket workers perform a substantial amount of manual material handling (MMH), including lifting, carrying, pushing and pulling.

Our research group recently found that full-time supermarket workers from a Danish medium-sized supermarket chain handled an average of 1200 kg of goods during a full workday, with some workers handling up to six tons daily^23^. The daily workload was associated with higher pain intensity in the low-back the following day in an exposure–response fashion, and the pain accumulated during consecutive workdays. These data indicate the large magnitude of manual material handling among supermarket workers as well as the negative consequences in terms of short-term pain development^23^. However, the study did not address potential differences in workload between specific departments and common work tasks that would enable guided preventive action in the supermarket industry.

Studies suggest that the prevalence of MSD^13,15–19^ and physical work demands^13,17–19,22^ varies between different supermarket departments. While most earlier studies have been focusing solely on the repetitive physical work of cashier work^24,25^, the present study focused on the physical work demands during supermarket stocking, including the transportation of goods from the storage rooms into the shopping area, where the goods are rearranged and/or stocked onto the shelves. Hitherto, the available studies assessing the physical work demands of supermarket work are predominantly based on qualitative interviews of retailers^26^ and observational methods^13,14,17,18^, with only few technical field measurements available (i.e. electromyography (*EMG*))^19,22,27,28^. While every method contains both strengths and limitations, technical field measurements, e.g. EMG, offer objective quantitative estimations of muscular workload^22,29–32^, which decrease the inherent biases associated with more subjective assessments of physical work demands^33,34^. Due to the enormous efforts required to collect and analyze such data, these studies are scarce and of inadequate level of detail to guide preventive initiatives in the supermarket sector^19,27^. Further, more recent studies are needed within the supermarket sector since the physical work demands may have changed over time due to changes in store design, technical assistive devices, flow of customers and goods^17^. Our research group recently reported a comprehensive assessment of the physical work demands during stocking activities among supermarket workers^22,28^. However, while these studies were carried out in a supermarket setting and closely mimicked real-life stocking work, they were performed under standardized conditions where workers were instructed what to lift and when to lift as it is normally done in the laboratory. An alternative approach is to measure what they actually do during un-restricted stocking work to capture real-life working conditions.

Therefore, the present field study applied combined surface electromyography (*sEMG*) and video recordings to estimate the muscular workload in the low-back and neck/shoulder muscles during un-restricted stocking activities in various store departments in five supermarket chains. We hypothesized that muscular workload vary between different types of supermarket chains, departments and work tasks.

## Material and methods

### Study design and setting

In accordance with guidelines for the reporting of observational studies in epidemiology (STROBE)^35^, this cross-sectional study reports the results from technical field measurements of EMG-derived myoelectric activity among Danish supermarket workers (n = 75). Throughout the paper, myoelectric activity will be termed ‘muscular workload’.

Collaborators from The Danish Chamber of Commerce were responsible for the recruitment of supermarket chains. Thereafter, chain representatives contacted their employees to find volunteers. This process began in 2018. Five supermarket chains of different types, including small discount stores, supermarkets, and hypermarkets (activity classes 47.1 and 47.19^36^) agreed to participate, from which around 15 workers from each chain volunteered. Chain A is characterized as a supermarket, Chain B is a hypermarket, whereas Chain C, D and E are discount stores. These different types of supermarket chains vary in total number of stores (number of stores ranging from 102 to 507) and average store sizes (average sales area per store ranging from 467 m^2^ to 2.156 m^2^). Data were collected between December 2018 and July 2019. All measurements and experimental procedures took place at the individual supermarket stores, predominantly during the morning shift, and lasted about three hours per participant, including instruction, instrumentation, technical measurements in the store, and finalizing the protocol (normalization, demounting the sensors, debriefing). As suggested by the supermarket chains, the morning shift was chosen as most of the stocking activities took place during this part of the workday.

### Participants

Written information about the research project was sent to potential participants prior to enrolment. Inclusion criteria included adult supermarket workers (≥ 18 years) working at least 30 h per week. Participants were excluded in case of severe cardiovascular disease, ambulatory measurements of blood pressure ≥ 160/100, and pregnancy^31^. The participants from each chain were generally evenly apportioned across three to five stores across Denmark.

All included participants (*n* = 75) were invited to complete an electronic questionnaire about basic characteristics, including work environment, lifestyle, and health. The questionnaire was completed before the day of testing by a personal questionnaire-link via e-mail, during preparation on the day of testing on the researcher’s laptop, or in the days following the technical measurements (via e-mail). The response rate was 89% (n = 67) for completing the entire questionnaire.

### Ethical approval

The study was approved by the Danish National Committee on Biomedical Research Ethics (H-3-2010-062). Adhering to the Helsinki Declaration, all participants received written and oral information about the study protocol and potential risks related to the measurements before providing oral and written informed consent. The National Research Centre for the Working Environment has a collective agreement with the Danish Data Protection Agency about data handling procedures to fulfill criteria for General Data Protection Regulation. The procedure for this study was approved by the in-house responsible person before initiating data collection. All data were de-identified and analyzed anonymously.

### Departments and work tasks

Based on procedures of previous studies, dialogues with industry representatives, preliminary field observations conducted in the five participating supermarket chains, and the categorization of video recordings, we defined six general store *departments* (Table 1) and the thirteen most common stocking *work tasks* that were representative for all the supermarket chains (Table 2). Thus, these generic work tasks were recurring across all five supermarket chains^22^, enabling comparisons of muscular workload not just between supermarket chains, but also between separate departments and work tasks.

**Table 1:** Definitions of departments.

Department	Typical goods
Fruit and vegetables (FV)	Apples, oranges, melons, bananas, cucumber, carrots, lemons, cabbage etc
Meat (M)	Cold cuts from roast beef, ham, chicken breast, salami, as well as larger meat cuts (tenderloin, fillet steak) and minced meat
Dairy (D)	Milk, butter, yoghurt, cheese, eggs
Frozen goods (F)	Ice cream, frozen vegetables and berries, ready-made food
Colonial (C)	Food and non-food with long shelf lives like flour, grains, sugar, coffee, tea, spices, tinned goods as well as toilet paper, kitchen roll, beer, wine, soda, candy, cleaning agents
Bread (B)	Rye bread, dark bread, white bread, sandwich bread

**Table 2:** Definitions of work tasks.

Work task	Definition
**Transport** Transport by pallet jack Transport by cart Transport by cage Push/pull of goods on floor	Transportation of parcels loaded on assistive devices or by pushing or pulling parcels on the floor
Single-item stocking	Stocking of smaller, lighter goods using one hand or two small goods in each hand, typically from parcels placed on assistive devices or the floor
**Two-handed lifts** Lifts from pallet jack Lifts from cart Lifts from cage Lifts from floor Lifts from pallet jack to cart Lift from cage to cart Lifts from pallet jack to cage	Heavier, two-handed lifts of parcels, either directly to the shelves or to another assistive device by which the parcel is transported or from where single items are stocked onto the shelves
Re-stocking	Re-arrangement of both light items (typically one-handed, like *Single-item stocking*) and heavier parcels (oftentimes two-handed, similar to ‘Two-handed lifts’) onto or from the shelves, backrooms or assistive devices

In some supermarket chains, workers specialize and hence stay in one department, whereas workers from other chains may do stocking work in all departments throughout a workday. However, most specialized workers had stocked in all departments throughout their employment or during previous employment, and all of them had been working in the supermarket sector for at least 6 months. During data collection, all participants performed 10–20 min of un-restricted stocking work in each of the five departments within the specific supermarket store they were employed, while technical measurements and video-recordings were carried out simultaneously by the principal investigators (SVS and RB). During the data analysis, these video recordings were used to categorize the stocking activities into the thirteen pre-determined work tasks. Consequently, we only assessed muscular workload during activities falling under the definitions of the pre-determined work tasks.

## Experimental design

The applied experimental procedure is almost identical to protocols previously used by our lab^22,31,37^. In brief, we combined sEMG measurements of the muscles of erector spinae longissimus and iliocostalis as well as trapezius descendens with video recordings. We chose these muscle groups because the low-back and neck/shoulder are among the most commonly affected body regions in terms of MSD among supermarket workers^13–20^.

More specifically, bipolar sEMG were recorded wirelessly (TeleMyo DTS Telemetry, Noraxon, AZ, USA) at a sampling rate of 1500 Hz and a bandwidth of 10–500 Hz, with the amplifier having a 16-bit A/D converter and a common mode rejection ratio > 100 dB. Before application of electrodes, the skin was shaved, cleaned and prepared using scrubbing gel (Acqua gel, Meditec, Parma, Italy) to reduce skin impedance. Afterwards, electrodes (Blue Sensor N-00-S, Ambu A/S, Ballerup, Denmark) were placed bilaterally on the selected muscles with an inter-electrode distance of two centimeters in accordance with SENIAM recommendations^38^. Electrodes and cables were fixed to the skin using stretch tape (Fixomull).

### Maximal voluntary isometric contractions

After mounting the equipment and before initiating the field measurements, an sEMG normalization procedure was performed for the erector spinae (in the lying Biering-Sørensen position^39,40^ and upper trapezius muscles^22,31^ consisting of maximal voluntary isometric contractions (MVIC)^41,42^. The latter was performed in a standing position with 90 degrees of arm abduction^37^. Subsequent to the technical measurements of the work tasks, additional MVICs were recorded for each muscle group before unmounting the equipment. The highest recorded value was chosen as the reference-MVIC for normalization. All raw surface EMG signals were digitally filtered using a Butterworth fourth-order high-pass filter (10 Hz cut-off frequency), full-wave rectified and smoothed using a root mean square (RMS) filter with a moving window of 500 ms. All trials were visually inspected and trials with non-physiological signal artefacts, i.e. spikes, gaps or low signal-to-noise ratio, were excluded. For each individual muscle and trial, the 95th percentile of the smoothed RMS signal was normalized (nRMS) to the maximal moving RMS (500-ms time constant) EMG amplitude obtained during the MVICs^43^. The nRMS values of the four erector spinae muscles (bilateral m. longissimus and iliocostalis) were merged^31^, resulting in a summed muscular workload for the low-back and neck/shoulders, respectively.

### Sample size

Naturally, a high variability in measured muscular workload is expected during field measurements due to variation in for instance work tasks and physical capacity of the workers^44^. Based on our previous EMG-based field measurements among workers performing manual material handling^44^, a minimum of 10 workers from each chain is required to ensure sufficient statistical power. Thus, we did not perform a new sample size calculation for the present study. The repeated measures design also entails a large statistical power to detect differences in muscular workload between different chains.

### Statistical analyses

The data were analyzed using linear mixed models with repeated measures (Proc Mixed, SAS version 9.4, SAS Institute, Cary, NC). Estimates are reported as least square means (LSM) with 95% confidence intervals of the 95th percentile rank of nRMS. Alpha levels below 0.05 were considered statistically significant.

All analyses were controlled for participant age (years, continuous variable) and sex (‘male’ or ‘female’, categorical variable). Chain- and department-specific muscular workload analyses were additionally controlled for chain, department, muscle as well as the interaction between chain and department. Analyses concerning muscular workload during specific work tasks were additionally controlled for chain, work task, muscle as well as the interaction between work task and chain. Sensitivity analyses showed that controlling for muscle strength (MVIC), body mass index and seniority did not add further to the model, and because some of these measures had missing data they were not included in the final model to retain a model without missing covariates.

## Results

Participant characteristics are presented in brief in Table 3 and in full in Supplementary Table 1. The mean age of the 75 participants was 30 years, with a slight majority of male participants (63%). Participants generally reported having good health and high physical work ability (Supplementary Table 1). However, MSDs of persistent nature (during the last three months) were prevalent among the participants, especially in the low-back (27% of the sample).

**Table 3:** Participant characteristics.

	*n*	Mean	SD	%
Age (years)	75	30	12	
Gender	75			
Women	27			37
Men	48			63
Height (cm)	67	175	10	
Weight (kg)	67	76	16	
BMI (kg/m^2^)	67	24.7	4.0	
Work experience within MMH (years)	67	9.7	10	

*n*  number, *SD * standard deviation, *%*  percentage.

### Stocking practices

The frequency of work tasks are reported in Table 4. *Re-stocking* was the most common work task, followed by *Single-item stocking*, *Lifts from pallet jack*, and *Lifts from cart* as the second, third, and fourth most common work tasks, respectively. The analyses suggested department-specific stocking practices, whereby some work tasks occurred more commonly in some departments compared to other departments (Chi-Square p < 0.0001). *Single-item stocking* and *Re-stocking* were the most or second most frequently reported work tasks in all five supermarket chains. Still, we observed different stocking practices between supermarket chains where some work tasks were more common in some chains than others (Chi-Square p < 0.0001). For instance, Chain C and D did *Lifts from pallet jack* more often than Chain A, B and E, who instead did *Lifts from cart* more often than Chain C and D. More specifically, some work tasks were more common in certain departments within specific chains compared to the same department in other chains (Chi-Square p < 0.0001).

**Table 4:** Stocking practices. Proportion (%) of all observed work tasks.

Work task	Proportion of work time					
	All	Chain A	Chain B	Chain C	Chain D	Chain E
Re-stocking	31.8	27.6	26.3	37.2	38.9	28.7
Single-item stocking	26.0	31.5	30.5	22.9	18.8	26.5
Lifts from pallet jack	12.6	3.4	2.4	32.0	19.3	6.0
Lifts from cart	8.6	9.6	12.2	1.1	5.1	15.1
Transport by cart	6.1	6.1	9.5	0.9	4.9	9.0
Push/pull of goods on floor	5.3	5.8	12.3	0.3	2.7	5.7
Lifts from cage	4.6	9.3	3.8	0.1	5.7	4.4
Transport by pallet jack	1.3	0.1	0.2	4.2	0.5	1.3
Lift from cage to cart	0.9	2.3	1.6	0.0	0.6	0.3
Lifts from pallet to cart	0.9	1.0	0.1	0.6	1.4	1.3
Transport by cage	0.9	2.3	0.9	0.3	0.4	0.5
Lifts from floor	0.8	0.6	0.1	0.4	1.7	1.2
Lifts from pallet jack to cage	0.2	0.5	0.1	0.2	0.0	0.0
Total	100	100	100	100	100	100

### Muscular workload

Chain-, department- and work task-specific peak muscular workload of the neck/shoulders and low-back are reported in Tables 5 and 6, respectively.

**Table 5:** Neck/shoulder muscular workload during supermarket stocking for chains, departments, and work tasks presented as least square means (LSM) with 95% confidence intervals of the 95th percentile rank of nRMS (% nRMS (95% CI)).

Chain		Department		Work task	
Chain E ^(1)^	19 (17–21)	M ^(1)^	17 (16–19) ^(3,4,5,6)^	Transport by cart ^(1)^	9 (8–10) ^(2,3,4,5,6,7,8,9,10,11)^
Chain D ^(2)^	19 (17–22)	F ^(2)^	18 (17–19) ^(3,4,5,6)^	Push/pull of goods on floor ^(2)^	12 (9–14) ^(1,5,6,7,8,9,10,11)^
Chain B ^(3)^	19 (17–22)	B ^(3)^	19 (18–20) ^(1,2,4,5,6)^	Transport by cage ^(3)^	13 (11–15) ^(1,5,6,7,8,9,10,11)^
Chain C ^(4)^	20 (18–22)	C ^(4)^	20 (19–21) ^(1,2,3,5,6)^	Transport by pallet jack ^(4)^	13 (11–16) ^(1,5,6,7,8,9,10,11)^
Chain A ^(5)^	20 (18–22)	D ^(5)^	21 (20–22) ^(1,2,3,4,6)^	Re-stocking ^(5)^	20 (19–21) ^(1,2,3,4,7,9,10,11)^
		FV ^(6)^	22 (21–23) ^(1,2,3,4,5)^	Single-item stocking ^(6)^	20 (19–21) ^(1,2,3,4,7,9,11)^
				Lifts from cart ^(7)^	21 (20–22) ^(1,2,3,4,5,6,11)^
				Lifts from floor ^(8)^	22 (19–24) ^(1,2,3,4)^
				Lifts from pallet jack ^(9)^	22 (20–23) ^(1,2,3,4,5,6)^
				Lifts from cage ^(10)^	22 (20–24) ^(1,2,3,4,5)^
				Lifts from pallet to cart ^(11)^	23 (21–24) ^(1,2,3,4,5,6,7)^

*n* = 75 supermarket workers.

*B*  bread, *C*  colonial, *D*  dairy, *F*  frozen goods, *FV*  fruit and vegetables, *M*  meat.

Significant differences (p < 0.05) between chains, departments and work tasks, respectively, are indicated with numbers in superscript.

**Table 6:** Low-back muscular workload during supermarket stocking for chains, departments, and work tasks presented as least square means (LSM) with 95% confidence intervals of the 95th percentile rank of nRMS (% nRMS (95% CI)).

Chain		Department		Work task	
Chain A ^(1)^	21 (18–24) ^(4,5)^	M ^(1)^	22 (21–24) ^(4,5,6)^	Transport by cart ^(1)^	20 (18–21) ^(4,5,6,7,8,9,10,11)^
Chain B ^(2)^	21 (18–25) ^(5)^	C ^(2)^	22 (21–24) ^(4,5,6)^	Transport by pallet jack ^(2)^	20 (18–22) ^(4,5,6,7,8,9,10,11)^
Chain E ^(3)^	25 (22–28)	F ^(3)^	22 (21–24) ^(4,5,6)^	Transport by cage ^(3)^	20 (19–22) ^(4,5,6,7,8,9,10,11)^
Chain D ^(4)^	26 (23–29) ^(1)^	B ^(4)^	25 (23–26) ^(1,2,3,5,6)^	Single-item stocking ^(4)^	22 (20–23) ^(1,2,3,5,6,7,8,9,10,11)^
Chain C ^(5)^	26 (23–29) ^(1,2)^	D ^(5)^	25 (24–27) ^(1,2,3,4,6)^	Re-stocking ^(5)^	24 (22–25) ^(1,2,3,4,7,8,9,10,11)^
		FV ^(6)^	27 (26–29) ^(1,2,3,4,5)^	Push/pull of goods on floor ^(6)^	24 (22–26) ^(1,2,3,4,7,8,9,10)^
				Lifts from cart ^(7)^	26 (25–28) ^(1,2,3,4,5,6,9,10,11)^
				Lifts from cage ^(8)^	27 (25–29) ^(1,2,3,4,5,6,11)^
				Lifts from pallet jack ^(9)^	28 (27–30) ^(1,2,3,4,5,6,7,11)^
				Lifts from pallet to cart ^(10)^	28 (27–30) ^(1,2,3,4,5,6,7,11)^
				Lifts from floor ^(11)^	36 (33–38) ^(1,2,3,4,5,6,7,8,9,10)^

n = 75 supermarket workers.

*B*  bread, *C*  colonial, *D*  dairy, *F*  frozen goods, *FV*  fruit and vegetables, *M*  meat.

Significant differences (p < 0.05) between conditions are indicated with numbers in superscript.

Overall, there were no significant differences in peak neck/shoulder workload between chains (~ 19–20% nRMS). However, peak muscular workload of the low-back (range: 21–26% nRMS) was significantly higher among workers from Chain C and D (26% nRMS, 95% CI: 23–29%) than among workers from Chain A (21% nRMS, 95% CI: 18–24%). In addition, workers from Chain B (21% nRMS, 95% CI: 18–25%) demonstrated significantly lower peak low-back muscular workload than workers from Chain C (26% nRMS, 95% CI: 23–29%). No other differences between chains were found.

There were small, but significant differences in neck/shoulder workload between all departments, except between the Meat (17% nRMS, 95% CI: 16–19%) and the Frozen Goods departments (18% nRMS, 95% CI: 17–19%), which were the departments with the lowest neck/shoulder workload. The Fruit & Vegetables (22% nRMS, 95% CI: 21–23%) department showed the highest neck/shoulder workload. With regards to low-back workload, there were no differences between the three departments with the lowest muscular workload, i.e. the Meat, Colonial and the Frozen Goods departments (22% nRMS, 95% CI: 21–24). In accordance with neck/shoulder workload, however, low-back workload in the Fruit & Vegetables department was significantly higher than in all other departments (27% nRMS, 95% CI: 26–29). There was substantial variation in department-specific workload between chains, with more marked differences between chains in low-back workload in the most physically demanding departments (see Supplementary Table 2). Specifically, the low-back workload in the Fruit & Vegetables departments ranged from 23–24% nRMS in Chain B and Chain A to 28–32% nRMS in the same department in Chain E, D and C.

In terms of work tasks, ‘Transport’ tasks were associated with the lowest workload in both the neck/shoulder and low-back muscles, whereas the typically heavier ‘Two-handed lifts’ were associated with highest neck/shoulder and low-back muscular workloads. Work tasks performed at low heights without technical aids, such as from the ground floor, specifically *Push/pull of goods/parcels on floor* (24% nRMS, 95% CI: 22–26) and especially *Lifts from floor* (36% nRMS, 95% CI: 33–38), were associated with significantly higher low-back workload than comparable work tasks carried out at higher heights by use of technical assistive devices, i.e. *Transport by cart* (20% nRMS, 95% CI: 18–21) or *Lifts from cart* (26% nRMS, 95% CI: 25–28).

## Discussion

This is the largest study to date applying technical measurements to systematically quantify the muscular workload during un-restricted supermarket stocking. Our results demonstrate differences in muscular workload—especially of the low-back—between different supermarket chains, departments as well as specific work tasks. This knowledge is of practical relevance for work environment professionals and local company managers aiming to reduce physical workload among supermarket workers.

### Comparison to previous studies

In the present study, 27% of the supermarket workers reported persistent LBP during the last three months, and around 60% of the participants had experienced LBP intensity ≥ 3 on a scale from 0 to 10 within the last week, a threshold level of LBP intensity that has been associated with work limitations^45^. Our study also showed that the low-back muscles (chain mean between nRMS 21–26%) generally seemed to work at the highest peak workloads during supermarket stocking compared to the neck/shoulder muscles (chain nRMS 19–20%). Previous EMG-based studies comparing workload of different muscles among supermarket workers have also reported highest workload in the low-back^22,27,46^. Moreover, some supermarket stocking tasks have recently been reported to exceed well-known tolerance limits for compression and shear forces in the lumbar spine^28^. These findings altogether suggest that preventative focus should be targeted at reducing physical work demands of especially the low-back muscles.

Our results show that the fruit and vegetables department is the most physically demanding department in supermarkets, both in terms of neck/shoulder and low-back workload. This finding aligns with our participants’ own experiences as well as our observations of the extent and magnitude of loads typically handled in this department, as also observed previously^20^. For instance, a common work task in this department is stocking of heavy parcels of bananas ranging in weight from 17 to 22 kilos. As reported by Skals and colleagues, handling of fruit and vegetables like bananas and cucumbers (in addition to milk in the dairy department) is associated with extraordinarily high peak muscle activity as well as compressive and shear forces in the low-back compared to stocking tasks in other departments^22,28^.

Direct comparison to other studies investigating department-specific physical workload is challenging due to different assessment methods and inconsistencies in the descriptions of the included functional departments^13,17–19,22^. Violante and colleagues ranked each supermarket departments by their ‘biomechanical risk’ based on qualitative observation of back postures and manual lifting actions^18^. In accordance with our results, the fruit and vegetables department ranked highest. Notably, the fruit and vegetables department have previously been reported as topping the list of department-specific prevalence of MSD^17,18^, which altogether indicates that work environment professionals and local managers should focus on reducing the physical workload in the fruit and vegetables department, specifically. Furthermore, we found significant variation in department-specific workload between chains, especially in the departments associated with the highest low-back workload. As an example, there were particularly large variation in low-back workload of the fruit and vegetables department between chains, ranging from ~ 23% to 31% nRMS. This could suggest that muscular workload in the supermarket sector could be reduced if inter-chain cooperation and exchange of experience and knowledge concerning good practice were prioritized. This may require a systematic approach, e.g. through workshops and in general close collaboration between work environment professionals of the different chains.

### Practical implications

A high physical workload is a known risk factor for MSD^1–4^. Hence, evaluating muscular workload with technical measurements during actual work conditions can be an important tool in the risk assessment of different work tasks. Our results corroborate with previous studies demonstrating higher physical workload with increased load weight^47–49^ granted that lighter work tasks, such as *Single-item stocking* or *Transport by cart*, generally demonstrated lower peak workload than work tasks typically associated with heavier parcels (and thus typically two-handed lifts), especially with regards to the low-back. Reducing the weight of the heaviest parcels (bananas, melons, cucumbers) thus seems imperative in terms of lowering the physical workload of supermarket stockers^22,28^. In accordance, LBP intensity have been shown to increase after workdays with higher compared to lower workload, after workdays compared to non-workdays, and after cumulated consecutive workdays among Danish supermarket workers^23^. These findings underscore the high potential of prioritizing workload management on both a daily and weekly basis.

The lower low-back workload when using technical aids (e.g. *Transport by cart* or *Lifts from cart*) further underscore the value of using technical assistive devices, particularly pertaining to peak workload of the back extensor muscles. More specifically, using technical assistive devices (a mobile cart) during stocking reduced the workload among supermarket workers compared to not using a technical assistive device^27^. Likewise, reduced physical workload was also measured when using technical assistive devices among nurses^31^. Fortunately, though, our results show that low-height work tasks performed from or on the floor (*Push/pull of goods/parcels on floor* and *Lifts from floor*) are very rare across all five participating chains, ranging from 0.1 to 1.4% and 0.1 to 1.7% of work tasks, respectively. Technical assistive devices typically enable handling at higher heights and thus implicate less MMH at lower heights, which reduces low-back loading^50,51^. Since lifting height is an important parameter in terms of physical workload^22,28,46,48,49^, re-organizing the supermarket *shelf heights* to better fit the workers, e.g. removing or adjusting the lowest and highest shelves, may be worth pursuing in terms of reducing the physical workload and improve the working and lifting positions^22,28,46^. This has already been adopted by some Danish supermarket chains.

### Strengths and limitations

The current study has several methodological strengths and limitations.

The current study is the largest (n = 75) and most comprehensive to date to quantify the muscular workload of stocking work in supermarkets. The inclusion of several supermarket chains of different types and sizes allow for a tentative generalization to other supermarkets not participating in the current study. Instead of relying on self-reports and observational methods that are more prone to bias^34^, this study offers valid information on how load is distributed to different muscle groups during un-restricted supermarket stocking by using the EMG method^27,29,31,49^. Methodologically, the within-subject repeated measures design reduces inherent variability commonly observed when simply comparing two cross-sections, thereby increasing statistical power.

The present study as well as previous studies using EMG are limited by e.g. difficulty establishing a true maximum effort reference, signal dropout, cross-talk, and poor skin–electrode contact^52^. Through a careful methodological approach we intended to minimize these common limitations. It is important to note that the measured myoelectric activity is merely a precursor to actual muscle activation and hence does not equal force development one-to-one, especially not under dynamic contractions and fatiguing conditions^52,53^. However, it should be mentioned that most work tasks are performed at relative low velocities compared to e.g. sport activities. Furthermore, EMG amplitude during controlled dynamic submaximal lifting (dumbbells) at load ranges comparable to those observed in the present study shows a close and almost linear association with the load^54^. Nevertheless, the inherent methodological limitations of EMG should not be neglected. Importantly, not only high physical workload^1–4^, but also psychological and social factors in leisure and during working hours^19,55^ are related to the development of MSD. Furthermore, an evidence-based tolerance limit for nEMG activity associated with risk of developing MSD remains to be established. Hence, we cannot justify whether the reported values are high or hazardous per se. In addition, it is still unclear whether peak workloads—as presented here—or *accumulated workload* is more important in terms of development of MSD^56,57^.

Further, our results reflect a time-limited snapshot of the workday as we did not measure throughout a full workday, which could have provided a more representative picture of the actual physical workload. On the other hand, the vast majority of goods are stocked during the morning hours, where we predominantly collected the data, whereas the rest of the day—particularly in stores with low flow of customers—is typically characterized by on-going re-stocking of shelves. Thus, it is possible that the muscular workload data collected during the morning hours may be slightly overestimated compared to the rest of the working day. Conversely, fatigue may have accumulated throughout the workday, which may have yielded higher EMG values^52^. We acknowledge the risk of random variation in other potentially contributing factors, e.g. day-to-day variation in work tasks and busyness, differences in store design and individual differences in MMH techniques. There was a substantial between-subject variation in work experience within MMH, which may affect lifting technique and thus workload^58^. However, seniority did not add further to the statistical models and were hence not included in the final model. Differentiating between electronic and manual pallet jacks may have provided useful information with regards to utilization of these specific technical assistive devices. In addition, we did not use Bonferroni correction of p-values for multiple comparisons as this procedure is used to guard against statistical type 1 errors, but at the same time is overly conservative and usually results in an abundance of statistical type 2 errors, especially when using small datasets. Also, the Bonferroni correction implies that outcomes should be completely independent, whereas in the present study, we clearly expected that heavier stocking activities would be associated with higher EMG (and hence dependent). Thus, some statistical type 1 errors may exist in the present results. Finally, the applied method is time-consuming, relatively expensive, requires expertise, and holds the risk of altered behavior due to the awareness of being observed (The Hawthorne effect), even though the participants were encouraged by both the researchers and supermarket representatives to do their job as they normally would.

## Conclusion

Differences in muscular workload were found—especially for the low-back—between different supermarket chains, departments as well as specific work tasks. This should incite targeted action by work environment professionals and local company managers in order to reduce the physical work demands among supermarket stockers.

## Supplementary Information

Supplementary Tables.

## Data Availability

The datasets generated and analyzed during the current study are available from the corresponding author on reasonable request.
