# Supplementary material for: Technical field measurements of muscular workload during stocking activities in supermarkets: cross-sectional study
Source: Sci Rep. 2022 Jan 18;12:934. doi: 10.1038/s41598-022-04879-8 (PMC8766430; doi:10.1038/s41598-022-04879-8)
Supplement: Supplementary file 1 — Supplementary Tables. [file 41598_2022_4879_MOESM1_ESM.pdf]

Title: Technical field measurements of muscular workload during stocking activities in supermarkets: cross-sectional study

Author list: Sebastian Venge Skovlund, MSc, PhD stipendiate, Rúni Bláfoss, MSc, PhD stipendiate, Sebastian Skals, PhD, Markus Due Jakobsen, PhD, Lars Louis Andersen, PhD

Supplementary Table 1. Full participant characteristics.

		**Whole cohort**				**Chain A**				**Chain B**				**Chain C**				**Chain D**				**Chain E**		
	n	Mean	SD	%	*n*	Mean	SD	%	*n*	Mean	SD	%	*n*	Mean	SD	%	*n*	Mean	SD	%	*n*	Mean	SD	%
Age (years)	75	30	12		16	35	15		13	23	4		16	28	9		15	27	10		15	37	14	
Gender	75				16				13				16				15				15			
Women				37				63				8				38				33				
Men				63				38				92				63				67				
Height (cm)	67	175	10		14	172	10		10	181	10		16	174	9		14	178	10		13	175	12	
Weight (kg)	67	76	16		14	72	15		10	80	11		16	72	14		14	77	16		13	79	20	
Work experience within MMH		9.7	10.0		14	12.4	8.5		10	7.1	3.3		16	6.6	7.1		14	8.0	11.3		13	14.5	14.3	
Smoking	67				14				10				16				14				13			
Yes, daily				31				36				10				50				7				46
Yes, now and then				9				7				20				6				7				8
Ex-smoker				18				14				40				13				21				8
No, never				42				43				30				31				64				38
Physical activity during leisure (%)	67				14				10				16				14				13			
Mostly sedentary				18				29				0				19				14				23
Light exercise at least 4 h				51				57				30				44				43				77
Sports or heavy physical activity at least 4 h per week				18				7				40				25				21				
Training and competing regularly and several times a week				13				7				30				13				21				
**Work factors**																								
Busyness during typical/standard week (0-10)	67	6.7	1.7		14	6.6	1.8		10	6.2	1.5		16	7.4	1.3		14	6.0	2.1		13	6.9	1.5	
Physical exertion during lift of milk box (0-10, 15 steps)	67	5.8	3.1		14	5.3	2.3		10	4.9	2.7		16	6.9	3.1		14	6.9	3.6		13	4.2	2.9	
Physical exhaustion after typical workday (0-10)	67				14				10				16				14				13			
Not exhausted				3				0				0				6								8
A bit exhausted				28				29				40				13				36				31

Somewhat exhausted		40		43		50		38		36		38
Very exhausted		25		21		10		44		21		23
Completely exhausted		3		7		0		0		7		0
Mental exhaustion after typical workday (0-10)	67		14		10		16		14		13	
Not exhausted		3		7								8
A bit exhausted		25		14		40		13		43		23
Somewhat exhausted		39		29		40		44		36		46
Very exhausted		24		43		10		38		14		8
Completely exhausted		9		7		10		6		7		15
Well-rested the morning after a workday	67		14		10		16		14		13	
Almost always		16		14		20		0		21		31
Often		16		14		20		13		14		23
Sometimes		46		57		40		69		36		23
Seldomly		18		7		10		19		29		23
Never		3		7		10		0		0		0
Physical work ability	67		14		10		16		14		13	
Very good		39		21		70		50		21		38
Good		48		64		20		38		71		38
Fair		13		14		10		13		7		23
Poor		0		0		0		0		0		0
Very poor		0		0		0		0		0		0
Stressed during the past two weeks	67		14		10		16		14		13	
Always		1		0		10		0		0		0
Often		25		36		10		31		36		8
Sometimes		40		50		40		44		43		23
Seldomly		22		14		30		19		14		38
Never		10		0		10		6		7		31
Contradictory demands	67		14		10		16		14		13	
Always		3		7		0		0		0		8
Often		9		7		0		13		21		15
Sometimes		31		29		40		44		29		31
Seldomly		43		50		50		44		43		46

Never		13		7		10		0		7		
Job role certainty	67		14		10		16		14		13	
Always		51		50		50		44		43		69
Often		40		36		50		44		50		23
Sometimes		7		14		0		13		0		8
Seldomly		1		0		0				7		0
Never		0		0		0		0		0		0
Emotional distress during work	67		14		10		16		14		13	
Always		0		0		0		0		0		0
Often		13		14		10		19		14		8
Sometimes		28		43		10		38		21		23
Seldomly		34		36		40		25		50		23
Never		24		7		40		19		14		46
Influence at work	67		14		10		16		14		13	
Always		19		21		10		25		14		23
Often		39		29		30		31		64		38
Sometimes		24		29		20		25		14		31
Seldomly		15		21		30		19		7		0
Never		3		0		10		0		0		8
Need for very fast work	67		14		10		16		14		13	
Always		30		21		20		31		29		46
Often		46		50		50		50		50		31
Sometimes		19		29		20		19		21		8
Seldomly		4		0		10		0		0		15
Never		0		0		0		0		0		0
Belonging to work community	67		14		10		16		14		13	
Always		67		64		90		69		43		77
Often		25		29		10		25		43		15
Sometimes		4		0		0		6		14		0
Seldomly		1		0		0		0		0		8
Never		1		7		0		0		0		0
Superior's willingness to listen to problems	67		14		10		16		14		13	

Always				52				57				30				50				43				77
Often				30				21				40				31				36				23
Sometimes				12				14				20				13				14				0
Seldomly				6				7				10				6				7				0
Never				0				0				0				0				0				0
Pain intensity during the last week (0-10)	67				14				10				16				14				13			
Headache		2.4	2.5			1.6	2.1			1.9	2.3			3.0	2.6			2.9	2.6			2.5	3.1	
Neck		2.3	2.4			1.9	2.2			1.3	1.4			2.0	1.9			3.0	2.5			2.9	3.2	
Shoulders		2.5	2.5			1.9	2.2			1.6	2.0			2.2	2.3			3.6	2.4			3.0	3.1	
Upper back		2.1	2.3			1.4	2.1			1.0	1.2			2.4	2.0			3.1	2.3			2.5	3.2	
Low-back		3.4	2.6			3.3	2.2			2.4	2.4			3.4	2.8			4.3	2.5			3.2	3.0	
Elbows		0.6	1.5			0.1	0.5			0.4	1.0			0.6	1.5			1.1	1.7			0.8	2.0	
Hands/wrists		1.9	2.4			1.1	1.7			1.0	1.8			3.0	3.0			2.6	2.5			1.2	2.3	
Hips		1.3	2.3			0.2	0.6			0.7	1.6			1.4	2.3			2.4	2.7			1.5	2.9	
Knees		1.9	2.4			1.4	1.8			1.5	1.5			2.3	2.5			2.6	2.9			1.5	2.9	
Feet/ankles		2.4	2.6			3.4	3.0			1.0	1.3			2.1	2.4			3.7	2.6			1.5	2.2	
Chronic musculoskeletal pain ≥3 months duration (Yes)	67				14				10				16				14				13			
Headache				18				21				10				19				14				23
Neck				18				21				0				6				21				38
Shoulders				19				21				10				19				14				31
Upper back				18				14				10				25				14				23
Low-back				27				36				30				19				21				31
Elbows				3				0				0				6				0				8
Hands/wrists				18				0				10				38				21				15
Hips				16				7				10				13				36				15
Knees				25				14				30				31				29				23
Feet/ankles				30				43				10				31				29				31
MSD-related sickness absence the past year	67				14				10				16				14				13			
No				88				79				90				88				100				85
Yes, one workday				4				14				10				0				0				0
Yes, two to five workdays				6				0				0				13				0				15

Yes, more than 10 workdays				1				7				0				0				0				0
MSD-interference on work (0-10)	67	2.9	2.9		14	3.5	3.1		10	1.5	2.1		16	3.3	3.1		14.0	3.4	2.4		13	2.0	3.4	
MSD-interference on leisure-time activities (0-10)	67	2.9	3.3		14	3.0	3.4		10	2.2	3.1		16	3.3	3.3		14.0	3.3	3.2		13	2.5	3.8	
General health status	67				14				10				16								13			
Excellent				13				7				40				0				7				23
Quite good				42				43				50				50				29				38
Good				37				50				10				38				50				31
Not good				7				0				0				13				14				8
Poor				0				0				0				0				0				0

Supplementary Table 2.

Department-specific neck/shoulder and low-back peak muscular workload estimates per chain presented as least square means (LSM) with 95% confidence intervals of the 95th percentile rank of nRMS (% nRMS (95% CI)). Significant differences (p<0.05) between conditions are indicated with numbers in superscript.

**Neck/shoulder**			**Low-back**	
**B**			**B**	
Chain E (1)	18.3 (15.9-20.7)		Chain B (1)	20.9 (17.3-24.6) (3,4,5)
Chain A (2)	19.2 (16.9-21.4)		Chain A (2)	21.0 (17.9-24.1) (3,4,5)
Chain C (3)	19.2 (17.0-21.4)		Chain D (3)	26.7 (23.6-29.8) (1,2)
Chain B (4)	19.6 (17.0-22.3)		Chain E (4)	26.8 (23.5-30.0) (1,2)
Chain D (5)	19.8 (17.5-22.1)		Chain C (5)	27.3 (24.2-30.3) (1,2)
**FV**			**FV**	

Chain E [1]	19.7 (17.3-22.1) [4,5]		Chain B [1]	23.4 (19.8-27.1) [4,5]
Chain A [2]	20.6 (18.3-22.8)		Chain A [2]	23.6 (20.5-26.6) [3,4,5]
Chain D [3]	20.6 (18.3-22.9)		Chain E [3]	28.1 (24.8-31.3) [2]
Chain C [4]	23.6 (21.5-25.8) [1]		Chain D [4]	29.0 (25.8-32.1) [1,2]
Chain B [5]	23.6 (21.0-26.3) [1]		Chain C [5]	31.9 (28.9-34.9) [1,2]
**F**			**F**	
Chain E [1]	16.4 (14.0-18.8) [5]		Chain B [1]	19.7 (16.1-23.4) [5]
Chain C [2]	16.6 (14.4-18.8) [5]		Chain A [2]	21.2 (18.1-24.2)
Chain B [3]	17.4 (14.7-20.0)		Chain E [3]	23.0 (19.7-26.2)
Chain D [4]	17.8 (15.4-20.1)		Chain C [4]	23.4 (20.4-26.5)
Chain A [5]	20.5 (18.2-22.7) [1,2]		Chain D [5]	24.5 (21.3-27.6) [1]
**M**			**M**	
Chain E [1]	16.6 (14.3-19.0)		Chain B [2]	19.5 (15.8-23.2) [4,5]
Chain B [2]	16.8 (14.2-19.5)		Chain A [1]	19.9 (16.9-23.0) [4,5]
Chain A [3]	17.7 (15.5-20.0)		Chain E [3]	22.3 (19.1-25.5)
Chain C [4]	18.1 (15.9-20.3)		Chain C [4]	24.3 (21.3-27.4) [1,2]
Chain D [5]	18.2 (15.9-20.5)		Chain D [5]	24.9 (21.8-28.1) [1,2]
**C**			**C**	
Chain B [1]	17.7 (15.1-20.4) [5]		Chain A [1]	19.6 (16.5-22.6) [4,5]
Chain C [2]	18.9 (16.7-21.1)		Chain B [2]	21.0 (17.3-24.7)
Chain A [3]	19.7 (17.4-21.9)		Chain C [3]	21.9 (18.9-25.0)
Chain D [4]	19.9 (17.6-22.2)		Chain D [4]	24.4 (21.3-27.6) [1]

Chain E [5]	22.0 (19.7-24.4) [1]		Chain E [5]	24.4 (21.2-27.7) [1]
**D**			**D**	
Chain D [1]	20.0 (17.8-22.3)		Chain A [1]	23.1 (20.1-26.2)
Chain E [2]	20.1 (17.8-22.5)		Chain B [2]	24.0 (20.4-27.7)
Chain B [3]	21.2 (18.6-23.9)		Chain E [3]	24.8 (21.6-28.1)
Chain C [4]	21.8 (19.6-24.0)		Chain D [4]	26.2 (23.1-29.3)
Chain A [5]	22.9 (20.6-25.1)		Chain C [5]	27.4 (24.3-30.4)
